# Single-Reaction Multiplex Reverse Transcription PCR for Detection of Zika, Chikungunya, and Dengue Viruses

**DOI:** 10.3201/eid2207.160326

**Published:** 2016-07

**Authors:** Jesse J. Waggoner, Lionel Gresh, Alisha Mohamed-Hadley, Gabriela Ballesteros, Maria Jose Vargas Davila, Yolanda Tellez, Malaya K. Sahoo, Angel Balmaseda, Eva Harris, Benjamin A. Pinsky

**Affiliations:** Stanford University School of Medicine, Stanford, California, USA (J.J. Waggoner, A. Mohamed-Hadley, M.K. Sahoo, B.A. Pinsky);; Sustainable Sciences Institute, Managua, Nicaragua (L. Gresh);; Laboratory Centro Nacional de Diagnóstico y Referencia of the Ministry of Health, Managua (G. Ballesteros, M.J. Vargas Davila, Y. Tellez, A. Balmaseda);; School of Public Health of the University of California, Berkeley, Berkeley, California, USA (E. Harris)

**Keywords:** dengue virus, chikungunya virus, Zika virus, multiplex molecular diagnostics, real-time RT-PCR, dengue virus, viruses, vector-borne infections, arboviruses

## Abstract

Clinical manifestations of Zika virus, chikungunya virus, and dengue virus infections can be similar. To improve virus detection, streamline molecular workflow, and decrease test costs, we developed and evaluated a multiplex real-time reverse transcription PCR for these viruses.

Zika virus is a mosquitoborne flavivirus that, in 2015, spread throughout the tropical and subtropical regions of the Western Hemisphere. In January 2016, the first autochthonous cases of Zika fever were confirmed in Nicaragua (*1*). The diagnosis of human Zika virus infections is confounded by a nonspecific clinical presentation, which overlaps substantially with that of dengue virus (DENV) and chikungunya virus (CHIKV) (*2*,*3*) and by cross-reaction with DENV IgM and DENV nonstructural protein 1 in assays for Zika virus (*4*–*7*).

Molecular assays can detect and differentiate these 3 pathogens during the acute phase of illness. Although a number of molecular tests have been published for detecting DENV and CHIKV, only 2 Zika virus real-time reverse transcription PCRs (rRT-PCRs) have been reported and were characterized by using human specimens (*6*–*8*). These assays are run as individual reactions, and molecular testing for all 3 viruses, using established protocols, requires multiple reactions for a single patient sample (*6*,*9*,*10*). We describe a Zika virus rRT-PCR that was designed to be run in multiplex with published assays for pan-DENV and CHIKV detection (*11*,*12*). We then evaluated the single-reaction multiplex rRT-PCR for Zika virus, CHIKV, and DENV (referred to as the ZCD assay) by testing clinical samples from persons with suspected cases in Nicaragua.

## The Study

The Zika virus primers and probe (Technical Appendix Table 1) were designed by using all complete or nearly complete (>10,000 kb) Zika virus genome sequences available in GenBank (n = 21) accessed March 28, 2014). Target sequences were subsequently confirmed to match strains from the Americas. All rRT-PCR reactions were performed on an ABI 7500 instrument (Applied Biosystems, Foster City, CA, USA) by using 25-μL reactions of the SuperScript III Platinum One-Step qRT-PCR kit (Life Technologies, Carlsbad, CA, USA) and 5 μL of RNA template. Cycling conditions for the ZCD assay were as follows: 52°C for 15 min; 94°C for 2 min; 45 cycles at 94°C for 15 sec, 55°C for 20 sec (acquisition), and 68°C for 20 sec. Each run included a no-template control and positive controls for Zika virus, CHIKV, and DENV.

Linear range and lower limit of 95% detection (95% LLOD) for each target were determined as recommended. We determined linear range and 95% LLOD for each target as recommended (*13*; Technical Appendix). The linear range of the ZCD assay extended from 10^8^ to 10 copies/μL for Zika virus and DENV-3 and from 10^8^ to 100 copies/μL for DENV-1, −2, −4, and CHIKV. The 95% LLOD for each target, in copies/μL of eluate (5 μL added to each ZCD reaction), was as follows: Zika virus, 7.8; CHIKV, 13.2; DENV-1, 11.7; DENV-2, 13.5; DENV-3, 4.1; DENV-4, 10.5.

Assay exclusivity was established by testing genomic RNA from the following viruses: West Nile, Japanese encephalitis, tickborne encephalitis, yellow fever, Saint Louis encephalitis, o’nyong-nyong, Semliki Forest, Mayaro, Ross River, Getah, Barmah Fores, and Unas (*12*,*14*). No amplification was detected for any of these viruses.

De-identified serum samples, collected from Nicaraguan patients with suspected Zika virus, CHIKV, and/or DENV infections, were tested (Technical Appendix). We tested 216 samples by using the ZCD assay and the pan–DENV-CHIKV rRT-PCR, which is a validated duplex assay containing the DENV and CHIKV primers and probes used in the ZCD assay (*12*). Both assays were performed on an ABI7500 (Applied Biosystems) (Table 1). A total of 173 samples were positive for DENV alone (n = 25), CHIKV alone (n = 110), or both (n = 38). The ZCD assay and pan–DENV-CHIKV rRT-PCR showed very good agreement for DENV detection (κ = 0.907). Six of 8 discrepant samples were co-infected with DENV and CHIKV, and the 2 discrepant samples with DENV mono-infections had cycle threshold (C_t_) values of 41.34 and 42.25. The 2 assays demonstrated good agreement for CHIKV detection (κ = 0.662). C_t_ for the 35 CHIKV discrepant samples were reached significantly later (mean 39.8, SD ±1.5) than the 113 concordant samples (28.7, ±SD 9.7; p<0.0001).

**Table 1:** Comparison of DENV and CHIKV detection in the ZCD assay and pan–DENV-CHIKV rRT-PCR*

ZCD assay	pan–DENV-CHIKV rRT-PCR						
	DENV Detection				CHIKV Detection		
	Pos	Neg	Total		Pos	Neg	Total
Pos	55	5	60		113	23	136
Neg	3	153	156		12	68	80
Total	58	158	216		125	91	216

*CHIKV, chikungunya virus; DENV, dengue virus; neg, negative; pos, positive; rRT-PCR, real-time reverse transcription PCR; ZCD assay, single-reaction multiplex rRT-PCR for Zika virus, CHIKV, and DENV.

The first case of Zika virus infection in Nicaragua was detected with the ZCD assay during the assay comparison described above. After Zika virus identification, 133 consecutive samples were tested by using both the ZCD assay and a comparator Zika virus rRT-PCR targeting the capsid gene (*6*) (Table 2). When the comparator rRT-PCR was analyzed according to the published validation (C_t_ <38.5 defining a positive result), these assays demonstrated only moderate agreement (κ = 0.47), and Zika virus was detected in significantly more samples by using the ZCD assay (p<0.001). Of the 31 samples positive only for Zika virus in the ZCD assay, 22 (71%) produced a C_t_ (>38.5) that was reached later in the comparator Zika virus rRT-PCR. These 22 samples had mean C_t_ of 32.06 (SD ±2.45) in the ZCD assay and 42.09 (SD ±1.41) in the comparator Zika virus rRT-PCR. If all samples in the comparator rRT-PCR with C_t_ >38.5 were considered positive, the assays demonstrated very good agreement (κ = 0.81; Technical Appendix Table 2). Of the 56 Zika virus–positive samples in the ZCD assay, 39 were positive only for Zika virus, and 17 showed evidence of mixed infection: Zika virus–DENV (n = 3); Zika virus–CHIKV (n = 10 ), or Zika virus–CHIKV–DENV (n = 4).

**Table 2:** Comparison of Zika virus detection in the ZCD assay and the Zika virus comparator rRT-PCR*

ZCD assay	Zika virus rRT-PCR		
	Pos	Neg	Total
Pos	25	31†	56
Neg	1‡	76	77
Total	26	107	133

*CHIKV, chikungunya virus; C_t_, cycle threshold; DENV, dengue virus; neg, negative; pos, positive; rRT-PCR, real-time reverse transcription PCR; ZCD assay, single-reaction multiplex rRT-PCR for Zika virus, CHIKV, and DENV. †22 samples produced a late C_t_ in the comparator Zika virus rRT-PCR (C_t_ >38.5) ‡Sample was also pos for a DENV-CHIKV co-infection and tested pos for Zika virus when repeated in the ZCD assay.

## Conclusions

The ZCD assay improved detection of Zika virus relative to the comparator rRT-PCR, and 31 samples were positive only for Zika virus by the ZCD assay when the comparator was interpreted as published (*6*). Notably, 22 (71%) of these 31 samples produced a late signal in the comparator Zika virus rRT-PCR (C_t_ >38.5), indicating that these most likely are true, late-positive results. Improved sensitivity for Zika virus is needed given the low viremia detected in clinical samples and the current lack of accurate alternative diagnostics, such as serology (*6*,*7*,*15*). Additionally, the ZCD assay identified 17 co-infections in Zika virus–positive patients. Although preliminary, these data provide evidence for the utility of a multiplex diagnostic test for these pathogens.

The performance of the ZCD assay for DENV detection was similar to that of the pan–DENV-CHIKV rRT-PCR, and the analytical sensitivity for CHIKV was similar in both assays (*12*). CHIKV detection in clinical samples in the ZCD assay and pan–DENV-CHIKV rRT-PCR demonstrated good agreement, although both assays contain the same CHIKV primers and probes. Discrepant samples all had C_t_ of >37.36, which correspond to 10 copies/μL of eluate and fall below the 95% LLOD. Although ZCD assay results for these CHIKV-positive samples were reproducible, the clinical significance of such low-level viremia in patients with suspected chikungunya fever is unclear and warrants further study.

A limitation to our study is the use of a single comparator Zika virus rRT-PCR. This assay was 1 of 2 rRT-PCRs developed for the 2007 Yap Island Zika virus strain (*6*). The second rRT-PCR, targeting the membrane gene, was evaluated for the current study but proved consistently less analytically sensitive. Therefore, performance of this second Zika virus assay most likely would not have affected result interpretation.

In conclusion, the single-reaction multiplex ZCD assay detected and differentiated Zika virus, CHIKV, and DENV. This assay should streamline molecular workflow and decrease test costs while improving detection of these 3 human arboviruses.

Technical Appendix Primers and probes included in the single-reaction multiplex real-time reverse transcription PCR (rRT-PCR) for Zika, chikungunya, and dengue viruses; assay development and analytical evaluation; statistical analysis; clinical evaluation; and comparison of Zika virus detection in the ZCD assay and comparator Zika virus rRT-PCR.
