# Supplementary material for: Single-Reaction Multiplex Reverse Transcription PCR for Detection of Zika, Chikungunya, and Dengue Viruses
Source: Emerg Infect Dis. 2016 Jul;22(7):1295–7. doi: 10.3201/eid2207.160326 (PMC4918162; doi:10.3201/eid2207.160326)
Supplement: Technical Appendix — Primers and probes included in the single-reaction multiplex real-time reverse transcription PCR (rRT-PCR) for Zika, chikungunya, and dengue viruses; assay development and analytical evaluation; statistical analysis; clinical evaluation; and comparison of Zika virus detection in the ZCD assay and comparator Zika virus rRT-PCR. [file 16-0326-Techapp-s1.pdf]

Article DOI: http://dx.doi.org/10.3201/eid2207.160326

Single-Reaction Multiplex Reverse Transcription PCR for Dection of Zika, Chikungunya, and Dengue Viruses

Technical Appendix

Assay Development and Analytical Evaluation

The single-reaction, multiplex real-time reverse transcription PCR (rRT-PCR) for Zika virus, chikungunya virs (CHIKV), and dengue virus (DENV) (referred to as the ZCD assay) was interpreted on the ABI7500 instrument (Applied Biosystems, Foster City, CA, USA) on the linear scale. Crossing thresholds were set manually for each target during analytical evaluation. Thresholds were set to cross amplification curves in the linear range of the assay at the start of the logarithmic phase of amplification. An exponential curve crossing the threshold was considered positive.

Linearity and lower limit of 95% detection (95% LLOD) studies were performed using quantitated, synthesized single-stranded DNA (ssDNA) that, individually, contained target sequences for each DENV serotype, CHIKV, and Zika virus. Linearity was evaluated by testing serial 10-fold dilutions from 8.0 $\log_{10}$ copies/µL to 1 copy/µL of eluate. Four replicates of each concentration were tested on a single run. The linear range was established by fitting a best-fit line to the data by regression analysis and included the range where the R^2 value for this line was ≥0.99. To establish the 95% LLOD, 10 replicates of five 2-fold dilutions extending from, and including, the lower limit of the linear range were tested on a single run. The 95% LLOD was then calculated using probit analysis.

All ZCD assay testing was performed in the molecular diagnostics laboratory at the Centro Nacional de Diagnóstico y Referencia (Managua, Nicaragua). To limit the possibility for contamination, this laboratory has dedicated areas for master mix preparation, nucleic acid extraction and assay set-up, and RT-PCR performance. Additionally, each run of the ZCD assay,

pan-DENV-CHIKV rRT-PCR, and Ziva virus comparator rRT-PCR included a negative control (water). The negative control was negative on all runs performed.

Statistical Analysis

The 95% LLOD was calculated by probit analysis using SPSS software (IBM, Armonk, NY, USA). Basic statistics were performed using Excel (Microsoft Corp, Redmond, WA, USA). Mean cycle threshold (C_t) values were compared by using Welch's *t* test. Fisher exact tests, κ statistics, and Welch's *t* tests were performed using GraphPad software (GraphPad Software, Inc., LaJolla, CA).

Clinical Evaluation

De-identified serum samples were obtained from: 1) a cohort study of DENV, CHIKV and Zika virus transmission in children based in District II of Managua; 2) a hospital-based study of dengue and chikungunya severity based in the Infectious Diseases Ward of the Hospital Infantil Manuel de Jesús Rivera, also in Managua; and 3) the national surveillance system of reportable illnesses for the country of Nicaragua (*3*–*5*). Samples were extracted with the QIAamp Viral RNA Mini Kit (Qiagen, Hilden, Germany) by using 140 μL of serum and a 60-μL elution volume. Research protocols were approved by the Institutional Review Boards at the Nicaraguan Ministry of Health; the University of California, Berkeley; and Stanford University.

The comparator Ziva virus rRT-PCR, which was used to evaluate the clinical sensitivity of the ZCD assay for Zika virus, was a published rRT-PCR targeting the capsid gene (including the primers Zika virus 1086 and Zika virus 1162c and the probe Zika virus 1107-FAM) (*6*). Samples with a $C_t \leq 38.5$ in the Zika virus rRT-PCR were considered positive; samples with a C_t >38.5 were considered negative, as described in the initial publication (*6*).

The DENV and CHIKV components of the ZCD assay were compared with the pan-DENV-CHIKV rRT-PCR performed on the ABI7500 rather than the CFX96, as previously described, because the ABI instrument demonstrated improved sensitivity (*1*). C_t values on the CFX96 instrument were, on average, 3.91 cycles later than on the ABI7500, which resulted in a clinical sensitivity for the pan-DENV-CHIKV assay of only 45.2% on the CFX96 compared with

the ABI7500 (data not shown). These findings highlight the need to verify assay performance characteristics whenever a molecular test is implemented on a new real-time instrument.

References

1. Waggoner JJ, Ballesteros G, Gresh L, Mohamed-Hadley A, Tellez Y, Sahoo MK, et al. Clinical evaluation of a single-reaction real-time RT-PCR for pan-dengue and chikungunya virus detection. J Clin Virol. 2016;78:57–61. PubMed http://dx.doi.org/10.1016/j.jcv.2016.01.007

2. Waggoner JJ, Abeynayake J, Balassiano I, Lefterova M, Sahoo MK, Liu Y, et al. Multiplex nucleic acid amplification test for diagnosis of dengue fever, malaria, and leptospirosis. J Clin Microbiol. 2014;52:2011–8. PubMed http://dx.doi.org/10.1128/JCM.00341-14

3. Gutierrez G, Standish K, Narvaez F, Perez MA, Saborio S, Elizondo D, et al. Unusual dengue virus 3 epidemic in Nicaragua, 2009. PLoS Negl Trop Dis. 2011;5:e1394. PubMed http://dx.doi.org/10.1371/journal.pntd.0001394

4. Kuan G, Gordon A, Avilés W, Ortega O, Hammond SN, Elizondo D, et al. The Nicaraguan pediatric dengue cohort study: study design, methods, use of information technology, and extension to other infectious diseases. Am J Epidemiol. 2009;170:120–9. PubMed http://dx.doi.org/10.1093/aje/kwp092

5. Narvaez F, Gutierrez G, Pérez MA, Elizondo D, Nuñez A, Balmaseda A, et al. Evaluation of the traditional and revised WHO classifications of Dengue disease severity. PLoS Negl Trop Dis. 2011;5:e1397. PubMed http://dx.doi.org/10.1371/journal.pntd.0001397

6. Lanciotti RS, Kosoy OL, Laven JJ, Velez JO, Lambert AJ, Johnson AJ, et al. Genetic and serologic properties of Zika virus associated with an epidemic, Yap State, Micronesia, 2007. Emerg Infect Dis. 2008;14:1232–9. PubMed http://dx.doi.org/10.3201/eid1408.080287

Technical Appendix Table 1. Primers and probes included in the ZCD assay*

Primers and probe	Sequence (5′ → 3′)	Concentration†	Location‡
Zika virus			
Forward	CAGCTGGCATCATGAAGAAYC	300nM	7332–7352
Reverse 1	CACTTGTCCCATCTTCTTCTCC	300nM	7411–7432
Reverse 2	CACCTGTCCCATCTTTTTCTCC	300nM	7411–7432
Probe§	CYGTTGTGGATGGAATAGTGG	100nM	7355–7373
CHIKV			
Forward	CATCTGCACYCAAGTGTACCA	300nM	2578–2598
Reverse	GCGCATTTTGCCTTCGTAATG	300nM	2654–2674
Probe§	GCGGTGTACACTGCCTGTGACYGC	100nM	2614–2637
DENV			
-1, -2, -3 Forward	CAGATCTCTGATGAACAACCAACG	350nM	86–109
-2 Forward C→T	CAGATCTCTGATGAATAACCAACG	350nM	87–110
-3 Forward C→T	CAGATTTCTGATGAACAACCAACG	300nM	85–108
-4 Forward	GATCTCTGGAAAAATGAAC	450nM	81–99
-1, 3 Reverse	TTTGAGAATCTCTTCGCCAAC	300nM	179–199, 178–197†
-2 Reverse	AGTTGACACGCGGTTTCTCT	350nM	152–171
-2 Reverse A→G	AGTCGACACGCGGTTTCTCT	350nM	152–171
-4 Reverse	AGAATCTCTTCACCAACC	450nM	173–190
Probe A	CTCGCGCGTTTCAGCATAT	100nM	136–154
Probe B	CTCTCGCGTTTCAGCATAT	100nM	137–155
Probe C	CTCTCACGTTTCAGCATATTG	100nM	135–155
Probe D	CTCACGCGTTTCAGCATAT	100nM	135–153

*CHIKV, chikungunya virus; DENV, dengue virus; ZCD assay, single-reaction, multiplex real-time reverse transcription PCR for Zika virus, CHIKV, and DENV

†Concentration of each oligonucleotide in the final reaction mixture is provided. DENV and CHIKV primers and probes have been published previously (*1*,2).

‡Genomic locations are based on the following reference sequences: Zika virus strain MR766-NIID (GenBank accession no. LC002520.1), CHIKV strain S27–African prototype (GenBank accession no. AF369024.2), DENV-1 U.S./Hawaii/1944 (GenBank: EU848545.1), DENV-2 New Guinea C strain (GenBank accession no. AF038403.1), DENV-3 strain H87 (GenBank accession no. M93130.1), and DENV-4 strain H241 (GenBank accession no. AY947539.1). For the DENV 1, 3 reverse primer, positions are listed in the order DENV-1, DENV-3.

§5′ fluor (excitation and emission wavelengths) and 3′ quencher pairs were the following: ZIKV, Cal Fluor Orange 560 (538 nm and 559 nm) and BHQ-1; CHIKV, TAMRA (557 nm and 583 nm) and BHQ-2; DENV, FAM (495 nm and 520 nm) and BHQ-1. DENV probes are BHQplus probes (Biosearch Technologies, Petaluma, CA, USA).

Technical Appendix Table 2. Comparison of Zika virus detection in the ZCD assay and comparator Zika virus rRT-PCR if samples cycle threshold values >38.5 in the comparator rRT-PCR were considered positive

	Zika virus rRT-PCR		
ZCD Assay	Positive	Negative	Total
Positive	47	9	56
Negative	3*	74	77
Total	50	83	133

*For 2 of 3 patients, another pathogen was detected in the ZCD assay (DENV–CHIKV co-infection and CHIKV mono-infection). CHIKV, chikungunya virus; DENV, dengue virus; rRT-PCR, real-time reverse transcription PCR; ZCD assay, multiplex rRT-PCR for Zika virus, CHIKV, and DENV.
